# *TP53*-related signature for predicting prognosis and tumor microenvironment characteristics in bladder cancer: A multi-omics study

**DOI:** 10.3389/fgene.2022.1057302

**Published:** 2022-12-09

**Authors:** Yuting Tao, Xia Li, Yushan Zhang, Liangyu He, Qinchen Lu, Yaobang Wang, Lixin Pan, Zhenxing Wang, Chao Feng, Yuanliang Xie, Zhiyong Lai, Tianyu Li, Zhong Tang, Qiuyan Wang, Xi Wang

**Affiliations:** ^1^ Department of Biochemistry and Molecular Biology, School of Basic Medicine, Guangxi Medical University, Nanning, China; ^2^ Key Laboratory of Biological Molecular Medicine Research, Education Department of Guangxi Zhuang Autonomous Region, Guangxi Medical University, Nanning, China; ^3^ Center for Genomic and Personalized Medicine, Guangxi Medical University, Nanning, China; ^4^ Guangxi Key Laboratory for Genomic and Personalized Medicine, Guangxi Collaborative Innovation Center for Genomic and Personalized Medicine, Nanning, China; ^5^ Departments of Urology, The First Affiliated Hospital of Guangxi Medical University, Nanning, China; ^6^ Department of Urology, Affiliated Tumor Hospital of Guangxi Medical University, Nanning, China; ^7^ School of Information and Management, Guangxi Medical University, Nanning, China

**Keywords:** bladder cancer, TP53, immunosuppression, tumor microenvironment, multi-omics studies

## Abstract

**Background:** The tumor suppressor gene *TP53* is frequently mutated or inactivated in bladder cancer (BLCA), which is implicated in the pathogenesis of tumor. However, the cellular mechanisms of *TP53* mutations are complicated, yet well-defined, but their clinical prognostic value in the management of BLCA remains controversial. This study aimed to explore the role of *TP53* mutation in regulating the tumor microenvironment (TME), elucidate the effects of *TP53* activity on BLCA prognosis and immunotherapy response.

**Methods:** A *TP53*-related signature based on *TP53*-induced and *TP53*-repressed genes was used to construct a *TP53* activity-related score and classifier. The abundance of different immune cell types was determined using CIBERSORT to estimate immune cell infiltration. Moreover, the heterogeneity of the tumor immune microenvironment between the high and low *TP53* score groups was further evaluated using single-cell mass cytometry (CyTOF) and imaging mass cytometry (IMC). Moreover, pathway enrichment analysis was performed to explore the differential biological functions between tumor epithelial cells with high and low *TP53* activity scores. Finally, the receptor–ligand interactions between immune cells and tumor epithelial cells harboring distinct *TP53* activity were analyzed by single-cell RNA-sequencing.

**Results:** The *TP53* activity-related gene signature differentiated well between *TP53* functional retention and inactivation in BLCA. BLCA patients with low *TP53* scores had worse survival prognosis, more *TP53* mutations, higher grade, and stronger lymph node metastasis than those with high *TP53* scores. Additionally, CyTOF and IMC analyses revealed that BLCA patients with low *TP53* scores exhibited a potent immunosuppressive TME. Consistently, single-cell sequencing results showed that tumor epithelial cells with low *TP53* scores were significantly associated with high cell proliferation and stemness abilities and strongly interacted with immunosuppressive receptor–ligand pairs.

**Conclusion:** BLCA patients with low *TP53* scores have a worse prognosis and a more immunosuppressive TME. This *TP53* activity-related signature can serve as a potential prognostic signature for predicting the immune response, which may facilitate the development of new strategies for immunotherapy in BLCA.

## Introduction

Bladder cancer (BLCA) is the 10th most common cancer and one of the leading causes of cancer death worldwide. It is approximately four times more common in men than in women, and tobacco smoking is the main risk factor (Sung et al., 2021). Based on the tumor node metastasis classification, BLCA can be categorized as non-muscle invasive BLCA (NMIBC), accounting for approximately 75% of newly diagnosed BLCA cases, and muscle invasive BLCA (MIBC), characterized by rapid progression, metastasis, and poor prognosis after treatment (Allen et al., 2021). However, studies have identified molecular subtypes of both NMIBC and MIBC distinguished by different clinical and genetic characteristics, suggesting that BLCA has a high degree of heterogeneity (Minoli et al., 2020). Although the molecular subtypes established by different groups contribute to advancing the understanding of BLCA progression and potentially predicting the response to treatment, they do not consider the tumor microenvironment (TME), comprising cancer cells, various kinds of stromal cells, and surrounding extracellular matrix (ECM) (Hinshaw and Shevde, 2019). Cancer cells form a chronic inflammatory, immunosuppressive, and tumor-promoting environment by closely interacting with the ECM and stromal cells, causing tumor growth and metastasis (Wang et al., 2017). It has been reported that TME correlates with tumor progression, patient outcomes, and therapeutic responses (Pfannstiel et al., 2019). Further investigation of the TME will aid in developing personalized treatment strategies and improving clinical outcomes of patients with BLCA.

*TP53* is a tumor suppressor gene that can trigger cell apoptosis or induce cell cycle arrest after sensing cellular stress or DNA damage (Kastenhuber and Lowe, 2017). *TP53* mutations cause cell-cycle dysregulation that allows replication of damaged DNA, leading to uncontrolled cell proliferation and tumorigenesis (Marei et al., 2021). Compared to other solid tumors, urothelial carcinoma tends to have a high frequency of somatic mutations in *TP53*, especially for MIBC (inactivating mutations) (Audenet et al., 2018). Moreover, correlations between *TP53* mutation and more advanced stage, grade, and poor survival have been reported for BLCA (Dueñas et al., 2019; Wu et al., 2020). Emerging evidence shows that *TP53* also affects the TME by reprogramming its components. P53 (encoded by *TP53*) dysfunction in cancer-associated fibroblasts can promote tumor malignancy by increasing the secretion of chemokines and cytokines (Moskovits et al., 2006). In addition, TP53 affects the ECM by negatively regulating ECM metalloproteinase, which is beneficial for tumor progression, by enhancing the generation of several matrix metalloproteinases (Zhu et al., 2009). As it mediates the polarization of M2-type macrophages, strengthens antigen presentation of dendritic cells, and regulates expression of immune checkpoint molecules, TP53 is considered as a key factor in immunity (Marei et al., 2021; Shi and Jiang, 2021). However, few studies have discussed the relationship between *TP53* and TME in BLCA, and its impact on tumor progression and patient outcome remains unclear.

In this study, we aimed to construct a *TP53*-related score based on a *TP53* activity-related gene signature. Distinct clinicopathological and molecular features were found between the high and low *TP53* score groups. CIBERSORT was applied to estimate the infiltration of immune cells in the two groups, and the abundance of different immune cells was compared. The heterogeneity of the tumor immune microenvironment between high and low *TP53* score groups was evaluated using single-cell mass cytometry (CyTOF) and imaging mass cytometry (IMC). Furthermore, we explored the crosstalk between *TP53* high/low score tumor cells and immune cells (T and NK cells) by analyzing published BLCA single-cell RNA-sequencing (scRNA-seq) data. Taken together, our study advances the understanding of the effects of *TP53* on the TME and provides a theoretical basis for personalized treatment of patients with BLCA.

## Materials and methods

### Patients and samples

The tumor tissue samples of 52 patients with MIBC in the First Affiliated Hospital of Guangxi Medical University, From February 2018 to October 2019. None of the participants received any cancer treatment prior to admission, and the diagnosis of MIBC was confirmed by two experienced pathologists. The current research has been approved by the Medical Ethics Committee of Guangxi Medical University (20220159). All patients signed informed consent.

### Public datasets download

The gene expression files (RNA-seq) and corresponding clinical data were downloaded from The Gene Expression Omnibus (GEO, https://www.ncbi.nlm.nih.gov/geo/) database (GSE13507 and GSE32894 containing 165 and 224 primary carcinoma samples, respectively). This research complied with the data access rules and release principles of the database.

### RNA-seq

Total RNA of fifrty-two MIBC samples was extracted with Trizol reagent (Life Technologies Corporation, Carlsbad, CA, United States). After mRNA-purification and reverse transcription, RNA-seq library was constructed in line with the instruction of the Illumina TruSeq RNA Sample Preparation Kit (Illumina) and then was amplified by PCR (Huang et al., 2020).

RNA-seq analysis was carried out by Illumina NextSeq 500 platform (Illumina, San Diego, CA, United States). The detailed analysis procedures were as follows: FastQC was used to evaluate the quality control of raw sequencing data, including the purity and quantity of RNA and the RNA integrity number (RIN) value. Fastp was used to filter the original data, and then HISAT2 (Pertea et al., 2016) was used to compare the filtered data with human reference genome (HG38) to get SAM file. Then we used samtools to convert SAM file into BAM file, and conducted sorted bam file. StringTie (Pertea et al., 2015) is used to assemble aligned reads and predict expression levels. Then use the R package (DESeq2) an analysis of the difference between the data obtained, Padj <0.05 and | Fold change | > 1.5 were considered to be the differential genes (DEGs) in this study. The differentially expressed genes were annotated based on the Gene Ontology (GO) database and the outcome was described by GOplot. RNA-seq data for the samples described above are available at the GEO database (GSE).

For the public data set, we use the R package “GEOquery (2.58.0)” to download GSE13507 and GPL6102 platform data; also download GSE32894 and platform GPL6947 data. and platform GPL6947 data. We removed a probe corresponding to multiple genes and probes without corresponding genes. Multiple probes corresponding to a gene were averaged to obtain a unique gene matrix. Then use the R package “limma (3.46.0)” an analysis of the difference between the data obtained, Padj <0.05 and | Fold change | > 1.5 were considered to be the DEGs (Davis et al., 2007; Ritchie et al., 2015).

### Mutation analysis

This study identified common *TP53* mutation sites according to literature review (Katiyar et al., 2000; Kirk et al., 2005), and focused on hot spots. First, the extracted DNA samples of BLCA were sent to Shanghai Yihe Application Biotechnology Co., Ltd. The process includes library construction, data quality control, sequence alignment, SNP calling, mutation detection and filtering, mutation annotation, *etc.* Among them, the sequencing was performed on the IlluminaX10 sequencing platform, the sequencing mode was Paired end, and the sequencing read length was 2 × 150 bp. Mutation screening criteria: 1) Total sequencing depth at mutation site greater than 30; 2) The total depth ratio of variation is greater than 1%; 3) Q = −10lgP >10. The data after sequencing were statistically analyzed for gene mutation information, and the samples with *TP53* specific site mutations were defined as *TP53* mutation group.

### Construction of *TP53* score

Based on a published *TP53* activity-related signature (Cancer Genome Atlas Research Network, 2017). 20 TP53-induced genes and 10 TP53-repressed genes were ultimately used to establish the TP53 activity-related score, and single-sample gene set enrichment analysis (ssGSEA) algorithm was further used to evaluate the score of every patient. The corresponding *TP53* score of each sample was calculated by TP53-induced gene score minus TP53-repressed gene score. Score = S_induce_-S_repress_. The genes used for TP53 score included 30, of which 20 TP53-induced genes were DDB2, FAS, GADD45A, RPS27L, EDA2R, ACAD11, TRIM22, SPATA18, AEN, FDXR, MDM2, CDKN1A, PTCHD4, ZMAT3, PANK1, ALDH4A1, ESR1, RGCC, GADD45B, and PHLDA3. The 10 TP53-repressed genes were CCNB1, PLK1, EED, CDK1, EZH2, CCNB2, E2F3, MYBL2, FOXM1, and E2F2. These 30 genes for score were detectable in all datasets. Please check these gene symbols in Table 1.

**TABLE 1:** TP53-induced and repressed genes symbols.

Gene	Induce	Repress
1	DDB2	CCNB1
2	FAS	PLK1
3	GADD45A	EED
4	RPS27L	CDK1
5	EDA2R	EZH2
6	ACAD11	CCNB2
7	TRIM22	E2F3
8	SPATA18	MYBL2
9	AEN	FOXM1
10	FDXR	E2F2
11	MDM2	—
12	CDKN1A	—
13	PTCHD4	—
14	ZMAT3	—
15	PANK1	—
16	ALDH4A1	—
17	ESR1	—
18	RGCC	—
19	GADD45B	—
20	PHLDA3	—

### Mass cytometry

After surgery, fresh BLCA and its adjacent tissues were taken and placed in a pre-cooling buffer and transported through a refrigerator. Then, the tumor tissue was cut to 1 mm size, and the collagenase I (Gibco) solution was added for lysis. The gentleMACS (Miltenyi) tissue processor was used for processing. The 70 µm aperture cell filter was placed on the ice to filter. After centrifugation, the supernatant was removed, and 3 times volume of red blood cell lysate (Solarbio, Beijing, China) was added. After centrifugation, the supernatant was discarded, and the pre-cooled PBS was added to resuspension, and the broken red blood cells were removed by centrifugation. Finally, the single cell suspension was frozen for mass spectrometry detection. Some purified antibodies for surface staining were conjugated with MaxPAR antibody labeling kit (Fluidigm) to conform to manufacturer’s protocol. It is purchased directly from suppliers (Fluidigm, San Francisco, California, United States). All antibodies were shown in Supplementary Table S2. Each specimen takes 1.5 × 106 living cells and encodes with barcode reagent (Lai et al., 2015; Wang et al., 2021). Cell viability was measured with 5 mmol/L cisplatin (Fluidigm). After fixation and infiltration with 1× Fixi buffer (Fluidigm), the cells were stained with metal-coupled antibodies for 30 min at the same temperature. Subsequently, 1 ml of 125 nm cell-like embedding agent ir (191/193 Ir, fluidim) was added to each sample for the identification of cell events. Finally, the cells were resuspended with the standard EQ™ Four Element Calibration Beads solution and stored at 4°C for further detection. After standardization of Helios 2 CyTOF mass cytometry flow cytometry, cell samples were detected. The generated data were uploaded to Cytobank (https://www.cytobank.org/) after deconvolution, and the results were visualized by t-distribution random neighbor embedding (t-SNE) algorithm for further analysis.

### Imaging mass cytometry

The antibodies panel used in IMC was shown in Supplementary Table S3. Formalin-fixed paraffin-embedded slices (FFPE) for downstream analysis were also obtained from the Department of Pathology under the guideline approved by the Medical Ethics Committee of the First Affiliated Hospital of Guangxi Medical University. All slices were stored at 4°C before the experiment. Paraffin slides were incubated at 65°C for 2 h. Then, slides were deparaffinized with xylene (4 × 5 min) and rehydrated in descending series of ethanol (2 × 100%, 5 min each; 2 × 95%, 1 × 85%, 1 × 75%, 2 min each). Antigen retrieval was conducted by washing in PBS for 9 min and then boiling in a pressure cooker with Tris-EDTA buffer (pH 9.0, Sangon Biotech (Shanghai) Co., Ltd., China) for 5 min. Slices were cooled down at room temperature for 30 min before washing in PBS for 9 min. Slices were blocked with 3% BSA in DPBS at room temperature for 45 min after washing. Then, slices were incubated in a humidified chamber with a metal-conjugated antibody cocktail that diluted by DPBS solution contained 0.5% BSA at 4°C overnight. After a day of incubation, slices were counterstained for 30 min at room temperature with Cell-IDTM Intercalator-Ir (Fluidigm) which was dissolved by DPBS at a diluted concentration of 1:400, washed twice by DPBS containing 0.1% Triton-X (Thermo scientific, Waltham, MA, United Kingdom) and absolute DPBS, dried for 20 min at room temperature after washing. 1 mm 2 regions of interests (ROIs) were determined and detected by a Hyperion imaging mass cytometer (Fluidigm) based on Fluidigm’s standard procedures, with a frequency of 200 Hz and 1 µm step increment. Data was outputted as original MCD files and visualized by utilizing the MCDTM viewer (version 1.0) and files were exported from the viewer as tiff formats. In order to generate cell segmentation masks and extract the intensity of markers in the panel, the Cellprofiler (version 3.1.9) was used (Jones et al., 2008). For further analysis, tiff files were imported into histoCAT (version 1.73) (Schapiro et al., 2017). FlowSOM (version 2.0) and t-SNE algorithm were used to perform subset and dimension reduce. The interaction patterns of cell subset were revealed by running neighborhood analysis.

Next, we divided 100 ROI into three regions: tumor region, tumor-stromal boundary and stromal region. First, we divided each ROI into 400 small blocks, which were numbered from N1 to N400 in turn. Then compared with HE sections, the small blocks located in the tumor region, tumor-stromal boundary and stromal region were marked respectively. According to the cell coordinate information, the number of cells in the tumor region, tumor-stromal boundary and stromal region was counted respectively. Finally, we analyzed the neighborhood composition of subsets cells, which were significantly different in different regions between the two groups. We defined a cell as the center and all kinds of cells within 4 μm from its cell membrane as its neighbors.

### Single-cell data download and analysis and IMvigor 210 cohort

The single-cell RNA sequencing data of 8 BLCA tissues were obtained from the published literature (Chen et al., 2020). The original data were further analyzed after quality control. Simply put, the integrated data matrix is reduced by principal component analysis (PCA). Use the Findclusters function in Seurat (version:“4.0.1”, resolution parameter: 0.1) to identify 21 subsets and visualize them using the t-SNE diagram. EPCAM and CD3G were used to identify tumor epithelial cells and T cells. *TP53* activity and stemness-related gene set were scored by single-sample gene set enrichment analysis (ssGSEA) pathway enrichment, and gene set enrichment analysis (GSEA) pathway enrichment analysis of tumor epithelial cell subsets was performed. The ligand-receptor interaction between tumor epithelial cell subsets and T cell subsets was visualized by iTALK.

We downloaded the expression matrix and phenotypic files in identification of an immunotherapy-responsive molecular subtype of bladder cancer through the R package ‘IMvigor210CoreBiologies (1.0.0)’. We selected 195 bladder patients for follow-up analysis. IMvigor 210 cohort is the bladder cancer data after immunotherapy, recording the binary-response status and immunophenotype of patients after immunotherapy. Binary-response can be divided into CR (completed response)/PR (partial response) and SD (stable disease)/PD (progressive disease), and immunophenotype can be divided into: Immune infiltration, immune rejection, desert type. Gender, race, and subtype of cancer were also recorded.

### Statistics

SPSS 20.0 software, two-way Student’s t-test and Kruskal–Wallis rank-sum test were used to perform statistical analysis. Overall survival of patients was assessed using Kaplan-Meier analysis. *p* < 0.05 was considered statistically significant.

## Results

### Low *TP53* score is associated with poor prognosis and clinical features

**FIGURE 1:**
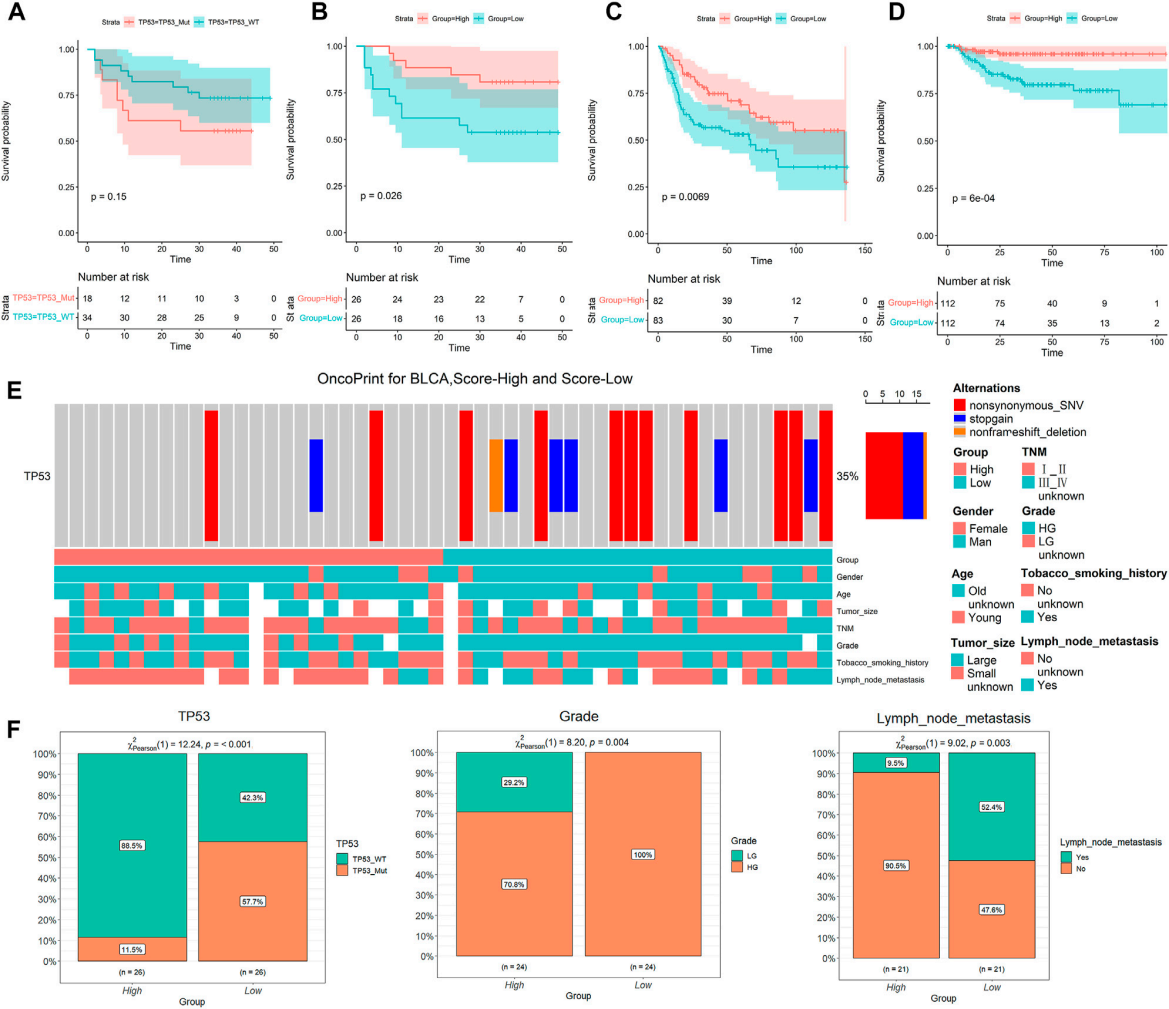
Low *TP53* score is associated with poor prognosis and clinical features. **(A)** Kaplan-Meier survival analysis for different *TP53* statuses. **(B–D)** Kaplan-Meier survival analysis of high and low TP53 score groups in our cohort, GSE13507 and GSE32894, respectively. **(E)** Overview of somatic mutations and clinical features in our cohort. **(F)** Chi-square analysis of high and low TP53 score groups with TP53 mutation, tumor grade and lymph node metastasis.

*TP53* status in cancer greatly influences patient survival, and patients with mutant *TP53* often have a poor prognosis (Rücker et al., 2012; Long et al., 2019; Wu et al., 2020). Therefore, we examined the association between the *TP53* mutant phenotype and survival rate in 52 patients with BLCA. It was found that the survival prognosis of patients could not be evaluated by TP53 mutation (Figure 1A). In addition, we downloaded data on *TP53* activity-related genes and scored the patients (Cancer Genome Atlas Research Network, 2017). Surprisingly, we found that the *TP53* activity score could better predict the survival prognosis of patients than the *TP53* mutation state, i.e., the prognosis of the low *TP53* score group (*TP53* inactivated group) was worse than that of the high *TP53* score group (Figure 1B). To further verify the reliability of the score in predicting prognosis, we performed *TP53* score and survival analysis on the external data set. Results revealed that low *TP53* score group had a worse prognosis than the high *TP53* score group (Figures 1C,D). Thus, *TP53* score can better reflect the prognosis of patients than *TP53* mutation.

We further explored the relationship between *TP53* scores and clinical characteristics of patients (Figure 1E). We found a significant positive correlation between a low *TP53* score and *TP53* mutation (Figure 1E), implying that the score conceivably reflects the state of *TP53* mutation. The low *TP53* scoring group also showed a significant correlation with high-grade cancer and lymph node metastasis (Figure 1F). These results suggest that BLCA patients with low *TP53* score have a worse survival prognosis.

### Immune cells and stem cell-related genes are highly expressed in low *TP53* score groups

**FIGURE 2:**
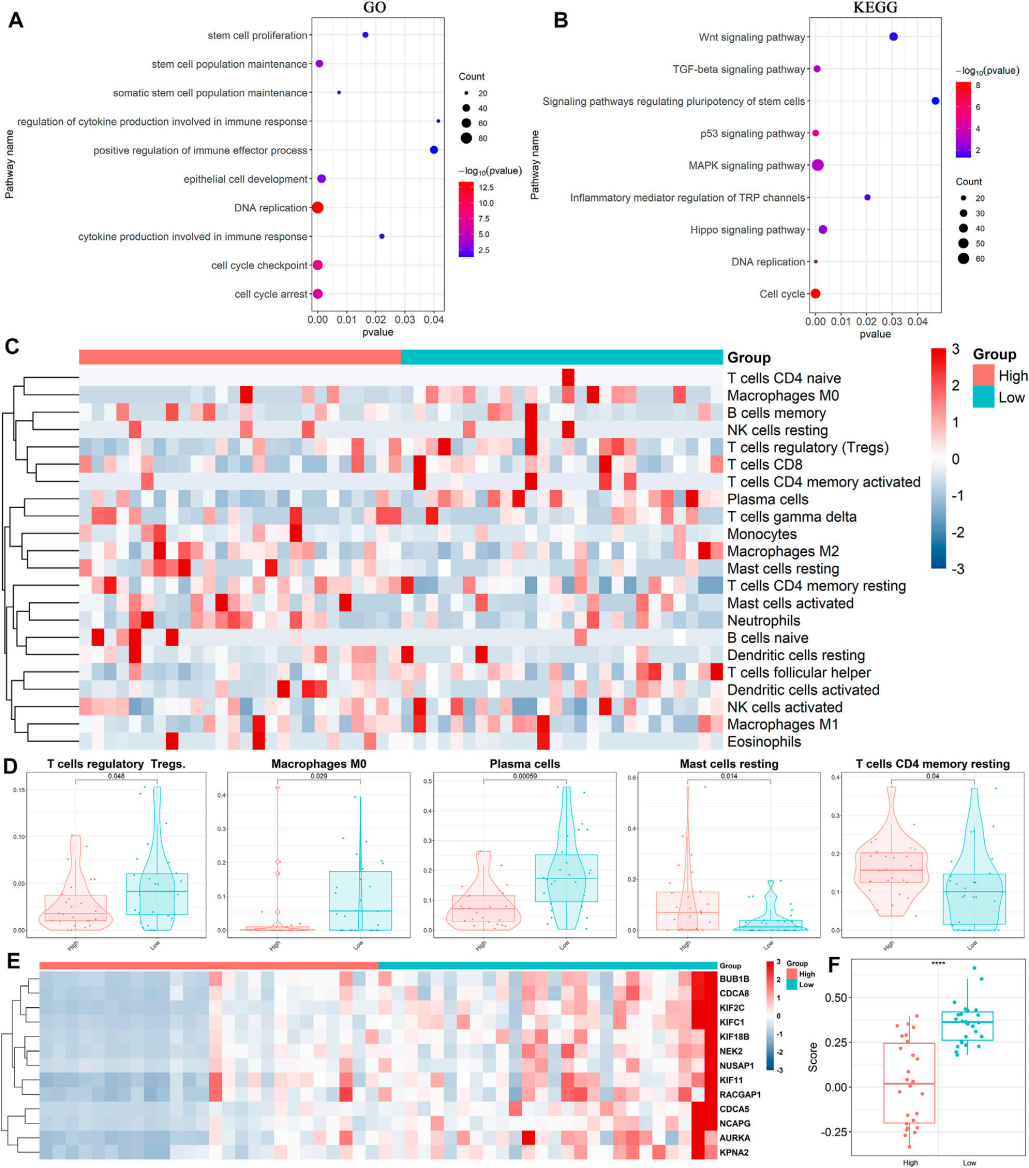
Expression of immune cells in high and low TP53 score groups. **(A,B)** Annotation of differentially expressed genes by Gene Ontology (GO) and Kyoto Encyclopedia of Genes and Genomes (KEGG). **(C,D)** Heatmap and box plots showed the expression of immune cells in high and low TP53 score groups. **(E,F)** Heatmap and box map showed the expression of stemness-related genes in high and low *TP53* score groups.

Based on the above results, we performed differential gene analysis in the high and low *TP53* scoring groups using data from GO and KEGG databases. The results showed that differential genes involved in stem cell proliferation, immune response regulation, and some signaling pathways, including Wnt, P53, and TGF-β, were enriched (Figures 2A,B). Next, we analyzed the gene sets of immune cell components in our BLCA cohort. Heatmaps displayed statistically significant differences in the composition of five immune cells between the high and low *TP53* score groups including T cell regulatory (Tregs), macrophage M0, plasma cells, Mast-cells-resting, and T-cells-CD4-memory-resting (Figures 2C,D; Supplementary Figure S1A,B). Moreover, the heatmap showed that stem cell-related gene set was highly expressed in the low *TP53* score group, which was verified using the ssGSEA algorithm (Figures 2E,F; Supplementary Figure S1C–F). These results suggest that immune cells and stem cell-related genes are highly expressed in patients with BLCA with low *TP53* scores.

### Patients with BLCA with low *TP53* score exhibit immunosuppressive TME

**FIGURE 3:**
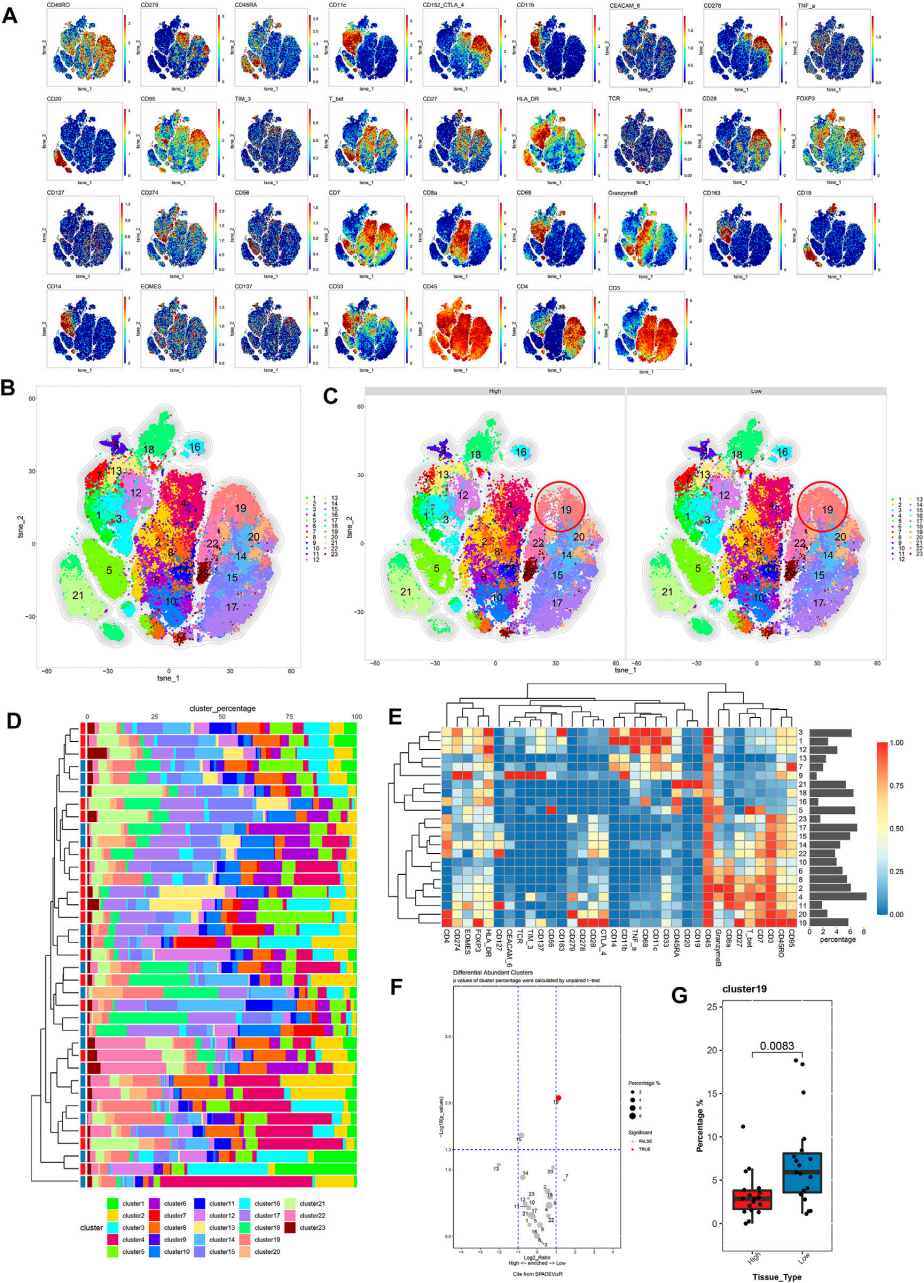
Immune cell composition and function description between high and low *TP53* score groups based on CyTOF. **(A)** t-SNE map of main immune markers expression. **(B,C)** High TP53 score group and low TP53 score group of BLCA patients immune microenvironment panorama. **(D)** Composition of cell subsets in high and low TP53 scoring groups. **(E)** Standardized heat map of immune marker expression. **(F)** Volcano map showed differential expression of tumor infiltrating immune cell subsets between high and low TP53 score groups. **(G)** There were significant differences in cell subsets between high and low *TP53* scoring groups.

To compare differences in the immune microenvironment between the high and low *TP53* score groups of patients with BLCA, We used CyTOF to analyze CD45 + cells obtained from 37 BLCA samples (high *TP53* score group = 18, low *TP53* score group = 19). A panel of 35 phenotypical and functional markers were co-stained (Supplementary Table S2) and t-SNE was used to identify distinct immune cell populations. CD45+ cells were divided into 23 cell subsets, including six CD4+ T cell (CD3+ and CD4+) subsets (14, 15, 17, 19, 20, 22, and 23), six CD8+ T cell (CD3+ and CD8+) subsets (2, 4, 6, 8, 10, and 11), one B cell (CD20+) subset (21), three mononuclear macrophage (CD14+) subsets (1, 3, and 7), two dendritic cell (CD11C+) subsets (12 and 13), three Treg (FoxP3+) subsets (16, 18, and 19), one myeloid granulocyte (CD11b+) subset (9), and one unknown subset (5) (Figures 3A–C,E). As shown in Figure 3D, the composition of various cell subsets of BLCA patients in the high and low *TP53* score groups. We subsequently compared the proportions of these cell subsets. It was found that cell subset 19 (Tregs) was enriched in the low *TP53* score group; the immunosuppressive Tregs in the low *TP53* score group were significantly more enriched compared with the high *TP53* score group (*p* < 0.05) (Figures 3F,G). Therefore, we speculated that patients with BLCA in the low *TP53* score group have an immunosuppressive microenvironment.

**FIGURE 4:**
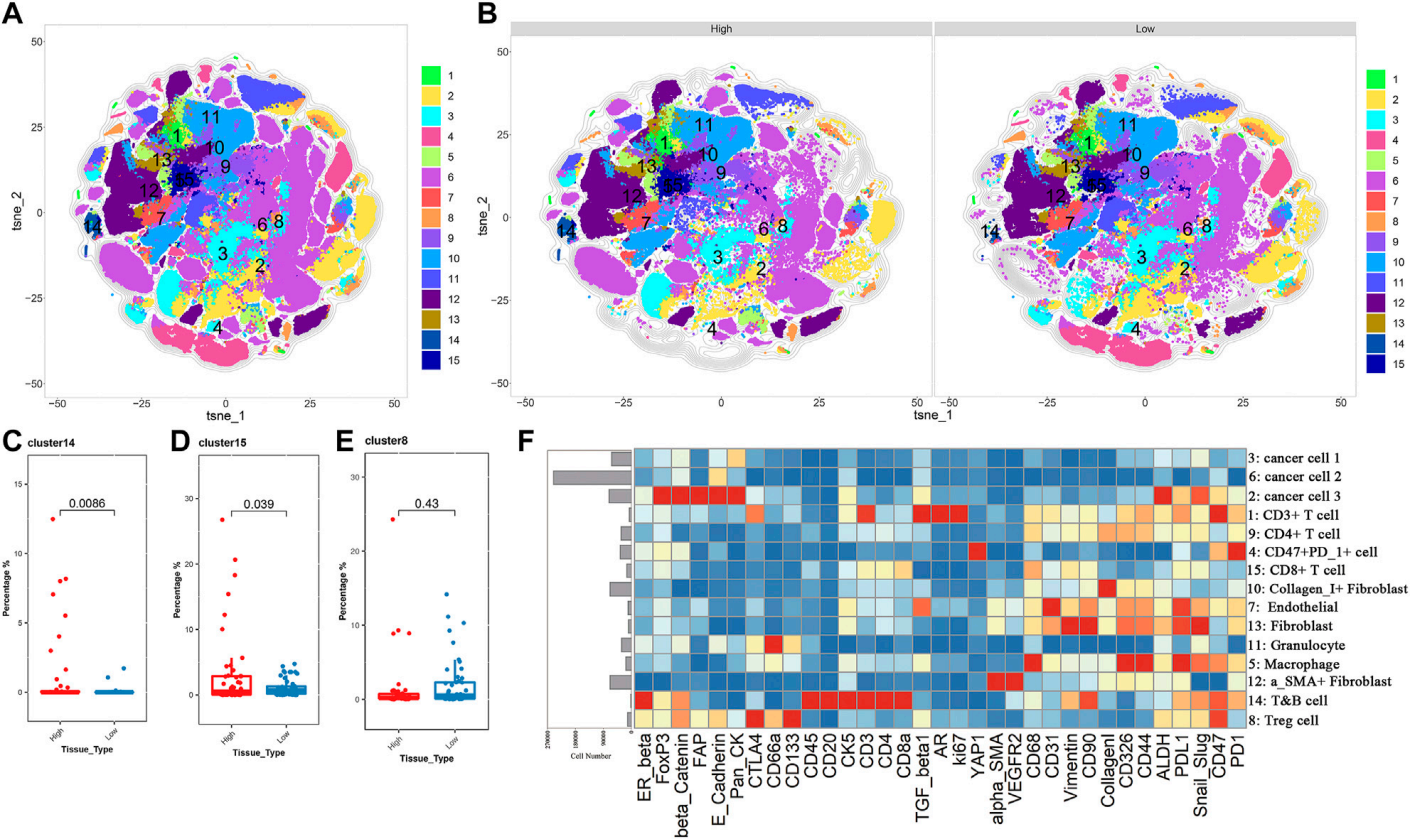
BLCA patients with low *TP53* score may exhibit immunosuppressive microenvironment. **(A,B)** T-SEN plots show the 15 cell subsets. **(C–E)** Box plots show the difference of subsets 8, 14, and 15 in the high and low TP53 score groups. **(F)** Heatmap of the expression of 33 markers after normalization of 15 immune cell subsets.

IMC is an extension of the mass cytometry flow technique that can detect more than 35 proteins on the same slice of tumor tissue and simultaneously capture and retain the spatial information of the cells in the region of interest (ROI). It is useful to identify the characteristic of cell subpopulations and cell interactions related to the diagnosis and treatment of tumors or the prognosis of patients (Chang et al., 2017). This technology has been applied to explore the characteristic and heterogeneity of tumor immune microenvironment. To further illustrate the difference in the TME between the low and high *TP53* score groups, the high-dimensional data of 100 ROIs (Region of Interest) contained in 22 BLAC IMC samples (low *TP53* score tissue (*N* = 11, 50 ROIs) and high *TP53* score tissue (*N* = 11, 50 ROIs) were used for dimensionality reduction clustering. Approximately 469084 cells were divided into 64 cell clusters using FlowSOM. Through the analysis of 64 cell subsets, we performed a secondary clustering to obtain 15 cell subsets, including 3 metaclusters of cancer cells, 1 metacluster of endothelial cells, 3 metaclusters of fibroblasts, and 8 metaclusters of immune cells (Figures 4A,B,F, Table S2). The tSNE map shows the distribution of 15 cell subsets between high *TP53* score tissue and low *TP53* score tissue (Figure 4B). We found that T&B cells and CD8 + T cells were significantly enriched in the tumor microenvironment with high *TP53* score tissue, while Tregs were not significantly different between the two groups (Figures 4C–E).

**FIGURE 5:**
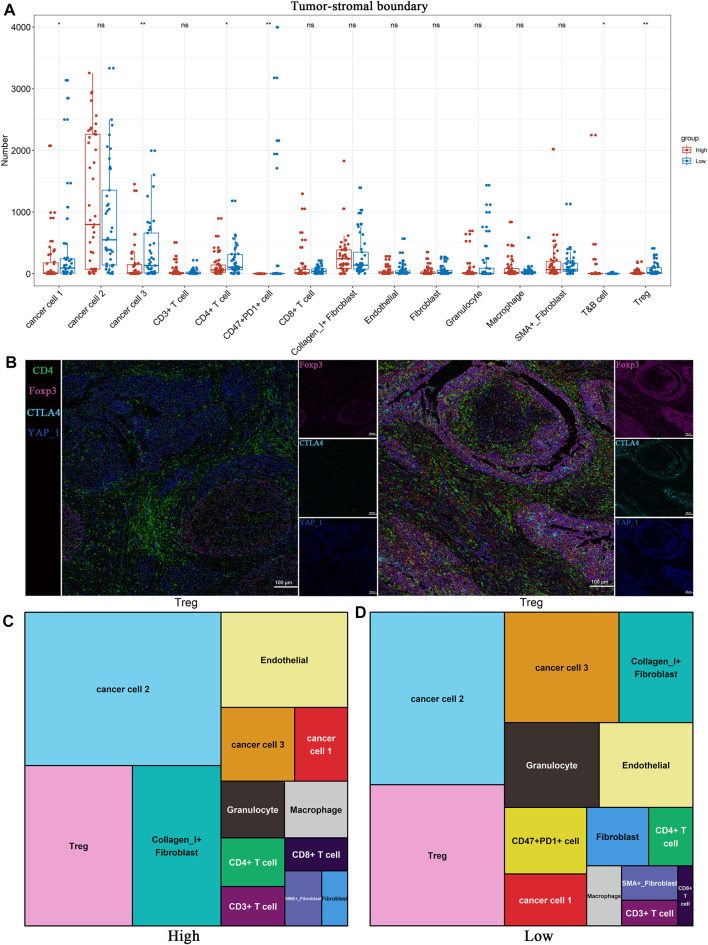
BLCA patients with low *TP53* score may exhibit immunosuppressive microenvironment. **(A)** Distribution of 15 cell subsets in Tumor-stromal boundary. **(B) **The representative IMC images showed the expression of immunosuppressive cell characteristic markers in the high and low TP53 score groups. **(C,D)** Tree diagrams shows the neighbors of Tregs subset in high and low TP53 score groups.

Considering the heterogeneity of the tumor microenvironment of BLCA, we divided each ROI of BLCA into three regions, including tumor region, tumor-stromal boundary and stromal region, for comparative analysis respectively. We found that Tregs were significantly enriched in the tumor-stromal boundary of low *TP53* score group (Figure 5A; Supplementary Figures S2, S3). As shown in Figure 5B, there are more Tregs in the tumor-stromal boundary of low *TP53* score tissue. The tumor-stromal boundary region is generally considered to be the frontier of tumor cell invasion and metastasis, which has an important influence on the occurrence and development of tumors. This region is enriched with more immunosuppressive-related cells. It is speculated that the tumor microenvironment in this region may inhibit normal immune cells to play its function, which is beneficial to the invasion and metastasis of BLCA cells. The various types of cells in the tumor microenvironment can not function without interacting with their surrounding neighbors, so we analyze the neighbor components of Tregs subsets in the tumor-stromal boundary (Figure 5C; Supplementary Figure S4). The results showed that more CD47 + PD1 + cell subsets, cancer cell 1, cancer cell 3 and granulocyte cell subsets were located near Tregs, indicating that patients with BLCA with low *TP53* score had a certain degree of immunosuppressive microenvironment. As shown in Figure 4F, cancer cell 1, a subpopulation of dedifferentiated BLCA cells, had significantly reduced expression of Pan_CK and the epithelial marker E_cadherin. Therefore, there are more dedifferentiated tumor cells in the tumor-stromal boundary of low *TP53* score group, which have greater invasion and metastasis potential (Figures 5C,D).

### High proliferation, low *TP53* score tumor epithelium have strong crosstalk with immunosuppressive cells

**FIGURE 6:**
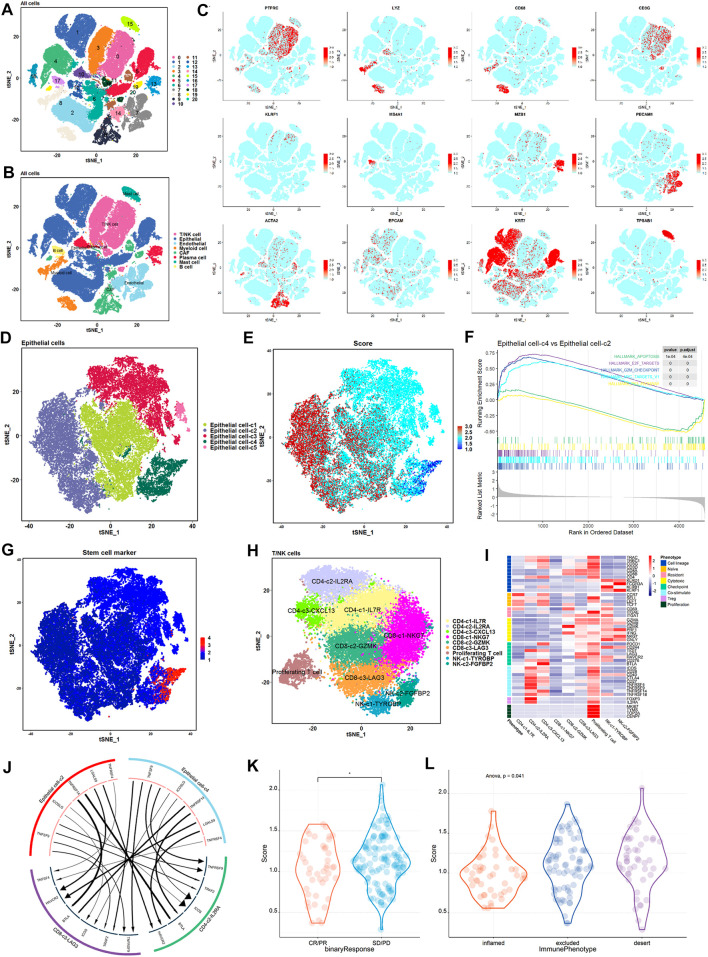
High proliferation, low *TP53* score tumor epithelium and immunosuppressive cells have stronger crosstalk. **(A)** t-SNE shows the annotation of cell subsets in BLCA. **(B)** Expression of major cell type marker genes. 8 main cell types: epithelial cells (EPCAM +/KRT7 +) ; endothelial cells (CD31 +) ; fibroblasts (ACTA2 +) ; b cells (CD20 +) ; myeloid cells (LYZ +) ; t/NK cells (CD3G +/KLRF1 +) ; mast cells (TPSAB1 +) ; plasma cells (MZB1 +). **(C)** t-SNE shows the annotation of eight main cell types in BLCA. **(D)** t-SNE showed epithelial cell subtypes in patients. Each subset is encoded in color according to cell type. **(E)** t-SNE showed the expression of TP53 active gene set in the epithelial cell subset of patients. **(F)** Epithelial cell-c4 enrichment pathway compared with epithelial cell-c2. **(G)** t-SNE showed the expression of BLCA stemness-related gene set in the epithelial cell subset of patients. **(H)** t-SNE showed the T and NK cell subtypes of patients. Each subset is encoded in color according to cell type. **(I)** Thermal map showed the expression of selected genomes in T/NK subtypes, including cell lines, naive, resident, toxicity, immune checkpoints, *etc.* **(J)** Receptor-ligand interactions are significantly enriched between epithelial cell-c4 and each immunosuppressive cell population. **(K)** Comparison of TP53 scores in patients with and without objective response in the IMvigor 210 cohort. **(L)** Comparison of TP53 scores for immunophenotypes of patients in the IMvigor 210 cohort.

To investigate the relationship between *TP53* activity-associated epithelial cell subsets and exhausted immune cells at the single-cell level, we analyzed scRNA-seq data from eight BLCA samples (Chen et al., 2020). We used Seurat for cell classification and marker gene identification and the t-SNE method to identify and visualize 20 subsets (Figure 6A). Nonimmune cells were mainly composed of endothelial cells (PECAM1 and ENG), fibroblasts (ACTA2 and RGS5), and epithelial cells (EPCAM and KRT19) (Figures 6B,C, and Supplementary Figure S5A). The identified immune cells included bone marrow-derived cells (LYZ and C1QB), T cells (CD3D, CD4, and CD8A), B cells (MS4A1 and CD79A), plasma cells (IGHG1 and MZB1), and NK cells (KLRF1 and KLRD1) (Figures 6B,C; Supplementary Figure S5A). In summary, 92029 cells from eight case samples were successfully identified as eight major cell types.

Next, we performed an unsupervised clustering of epithelial cells and obtained a *TP53* activity score to differentiate the epithelial subpopulations with different scores (Figures 6D,E). Among the five epithelial cell subsets, epithelial cell-c4 exhibited the lowest scores, whereas epithelial cell-c2 exhibited the highest scores (Figures 6D,E). Further pathway enrichment revealed that epithelial cell-c4 was significantly enriched in cell proliferation-related pathways, such as MYC targets, E2F targets and G2M checkpoint (Figure 6F). Stemness-related gene set expression was the highest in epithelial cell-c4 with a low *TP53* score and the lowest in epithelial cell-c2 with a high *TP53* score (Figure 6G). This suggests that the epithelial subsets with lower scores had a higher proliferative capacity than those with higher scores.

To further explore the interaction between epithelial cells and depleted T cells, we performed unsupervised clustering of T/NK cells, and classified T/NK cells into nine subsets based on associated marker genes, including three CD4 cell subsets (CD4-c1-IL7R, CD4-c2-IL2RA, and CD4-c3-CXCL13), three CD8 cell subsets (CD8-c1-NKG7, CD8-c2-GZMK, and CD8-c3-LAG3), one proliferating cell subset, and two NK cell subsets (NK-c1-TYROBP and NK-c2-FGFBP2) (Figures 6H,I). We used iTALK to analyze the interactions between epithelial subsets and exhausted immune cells (Figure 6J). We mainly focused on the difference in the ligand–receptor pairs between epithelial cell subsets with highest and lowest *TP53* score and immune cell subsets with the expression of exhausted surface markers IL2RA and LAG3. The results showed that expression of immunosuppressive ligands with the lowest score for epithelial cell-c4 was the strongest (Figure 6J).

Finally, to assess whether the *TP53* score can predict the efficiency immune checkpoint inhibitor therapy, we performed a *TP53* score on the IMvigor 210 cohort. After treatment, patients who achieved objective remission scored lower than those who did not (Figure 6K). Figure 6L shows significant differences in TP53 scores among the three groups of patients (Figure 6L). Taken together, patient score negatively correlated with the degree of immune cell infiltration and outcome after treatment with immune checkpoint inhibitors (Figures 6J,K; Supplementary Figure S5B). Thus, patients with low *TP53* scores may adapt more to immune checkpoint inhibitor therapy.

## Discussion

BLCA is characterized by multiple molecular and genetic alterations that together constitute the genotype, and thereby dictating tumor behavior. It is therefore reasonable that the compilation of genetic changes is a better indicator of clinical outcomes than a single gene. *TP53* is one of the most common mutations in BLCA, and BLCA patients with *TP53* mutations tend to present with a relatively poorer prognosis and higher grade of pathology than patients without *TP53* mutations (Ciccarese et al., 2017). As broadly known, *TP53* plays a central role in various key cellular functions related to cancer development, progression, and response to therapy (Olivier et al., 2010). For instance, *TP53* has been widely proposed as the “guardian of the genome” and “cell-cycle mediator,” as it responds to DNA damage, allowing DNA repair, facilitating cell apoptosis, and maintaining genomic integrity (Liu et al., 2019). In addition, *TP53* mutations in cancers can potentially affect immune cell recruitment and activity, resulting in various outcomes that may support or impede cancer progression (Blagih et al., 2020). Although previous studies indicated that TP53 mutation status does not necessarily correspond to the significant prognostic value and benefit of combined chemotherapy, these studies have some limitations; the proportion of cases in treatment in these studies was well below expectations, and, more importantly, was restricted to the genomic level and lacked comprehensive TP53 pathway analysis (Malats et al., 2005; Stadler et al., 2011). The underlying mechanism of *TP53* mutations in regulating the TME and prognosis of BLCA remains largely unknown. Hence, it is of great importance to explore the role of *TP53* mutation in regulating TME and further illustrate the relationship between *TP53* activity status and BC prognosis.

In this study, we used a surrogate method to evaluate *TP53* functional status based on the expression of *TP53* transcriptional targets. A *TP53* activity-related signature was established based on P53-induced genes and TP53-repressed genes (Cancer Genome Atlas Research Network, 2017) The corresponding *TP53* score (*TP53* classifier) was calculated by TP53-induced gene score minus TP53-repressed gene score (Table 1). Interestingly, this *TP53* activity-related gene signature differentiates well between *TP53* functional retention and inactivation in BLCA and provides a more accurate and useful method for determining TP53 clinical value than TP53 mutation status alone. Furthermore, we successfully verified the feasibility of the *TP53* activity-related signature, which can also predict overall survival with significant accuracy in independent GEO datasets of BLCA. Subsequently, functional enrichment analyses were performed to identify the potential biological functions regulated by *TP53* activity-related signatures. As expected, the results of GO and KEGG demonstrated that *TP53* activity-related signature was significantly associated with classical tumor-associated functions, including Wnt signaling and stem cell proliferation and maintenance signaling, and some immune-associated functions such as TGF-β signaling and cytokine production signaling were also enriched. In addition to the above analyses, CIBERSORT was used to evaluate the abundance of 22 immune cell types between the high- and low-*TP53* score groups. There was an obvious infiltration of immune cell types, including Tregs, M0 macrophages, plasma cells, and memory resting CD4 T cells, in the low *TP53* score group compared to the high *TP53* score group. Several studies have suggested that Tregs can exploit immunosuppressive functions by expressing a variety of immunosuppressive molecules and secreting inhibitory cytokines (Cai et al., 2019). M0 macrophages can be polarized to M1 or M2 *via* environmental signaling, implying an immunosuppressive role in cancer progression (Miao et al., 2017). The activation of mast cells not only produces the inhibitory cytokine IL-10 but is also essential for Treg-mediated immune tolerance (Huang et al., 2008). Moreover, a core set of 13 genes with stemness characteristics in BLCA was found to be significantly higher in the low *TP53* score group than in the high *TP53* score group (Pan et al., 2019). Taken together, these results indicate that the *TP53* activity-related signature is one of the risk factors leading to differences in BLCA prognosis, exhibiting immunosuppressive and stemness features.

With the rapid advancement of cutting-edge technologies, single-cell omics has enabled profiling of the TME at high dimension and single-cell levels. In this study, we initially conducted CyTOF, which combines mass spectrometry with flow cytometry and provides measurements of several simultaneous cellular parameters at single-cell resolution, significantly augmenting the ability to evaluate complicated heterogeneity of the TME (Tanner et al., 2013; Spitzer and Nolan, 2016). We used it to accurately divide the tumor-infiltrating immune cells into 23 cell subsets, and cell subset 19 with CD4^+^ and FoxP3+ expression was significantly enriched in the low *TP53* score group. Indeed, Foxp3-expressing Tregs, which are indispensable for preventing autoimmunity, also effectively suppress tumor immunity (Tanaka and Sakaguchi, 2019). Meanwhile, the infiltration of Tregs into tumor tissues is often associated with poor prognosis *via* the suppression of MMP2 in BLCA TME (Winerdal et al., 2018). In addition, tissue-based imaging technology IMC was further performed to investigate the cellular microenvironment and cell–cell interactions, which significantly contribute to the cell functional state (Hartmann and Bendall, 2020). Consistent with the afore mentioned previous findings, the low *TP53* score group exhibited a significant abundance of FoxP3+PD1+PDL1+ cells (cluster 1) in this study, indicating that the context of low *TP53* score group may be rendered as immune depletion. Notably, FOXP3 colocalizes with the immune checkpoint molecule CTLA4 and Hippo pathway downstream effector YAP1. According to previous studies, YAP activity is fundamental for stem cell maintenance, metabolism regulation, and tissue homeostasis (Piccolo et al., 2014; Koo and Guan, 2018). The recent discovery has identified YAP as an unexpected amplifier of FOXP3 expression and a Treg-reinforcing pathway with significant potential as an antitumor immunity therapeutic target (Ni et al., 2018). However, the microenvironmental cues and potential mechanisms governing CTLA4 and YAP1 colocalization warrant further investigation. Considering the spatial heterogeneity of the tumor microenvironment in bladder cancer, we also compared the cellular infiltration in different regions, including tumor region, tumor-stromal boundary, and stromal region. Interestingly, Tregs were significantly enriched in the tumor-stromal boundary of low *TP53* score group, while the tumor and stromal regions are not observed. As for cancer cells located in the boundary regions, they tend to exhibit invasive phenotype states and microenvironmental features (Bastola et al., 2020). The enrichment of Tregs was observed at the invasive tumor front, which may suggest the invasive and anti-tumor immunity potential. Recently, Sheng et al. (2022) modified an advanced analysis method for highly multiplexed pathological images based on cellular neighborhood. We also adopted their analytical strategy to calculate the complex interplay between Tregs. Intriguingly, another immunosuppressive cluster of CD47+PD-1+ cells showed strong interactions with Tregs in the low *TP53* score group (Chen et al., 2021), further confirming the hypothesis that an immunosuppressive TME exists in patients with BLCA with a low *TP53* score.

Advances in scRNA-seq have enabled the unprecedented molecular characterization of cell types possessing specific gene signatures (Xiong et al., 2020). In this study, we also downloaded an external scRNA-seq dataset and explored the relationship between *TP53*-related activity score between epithelial cell subsets and exhausted immune cells. We performed unsupervised clustering and identified five epithelial cell subsets with different *TP53* activity scores. Further pathway enrichment revealed that epithelial cell-c4 with the lowest *TP53* activity score was significantly enriched in E2F targets, MYC targets, G2M checkpoints, and other proliferation-related pathways compared to epithelial cell-c2 with the highest *TP53* activity score. There is evidence that high enrichment of G2M checkpoint and E2F target pathways are associated with *TP53* alteration and, more importantly, with cell proliferation characteristics, which implies the efficacy of *TP53* activity-related classifier and potential biological significance (Oshi et al., 2021). Moreover, epithelial cell-c4, with the lowest *TP53* activity score, responded to some immune-exhausted receptor-ligand pairs, including CD4-c2-IL2RA and CD8-c3-LAG3. The alpha subunit of the IL2 receptor (IL2RA) is a canonical marker of Tregs and has been implicated in immune suppression in cancer (deLeeuw et al., 2015). As such, lymphocyte activation gene 3 (LAG3) is expressed on multiple cell types, including CD4^+^ and CD8^+^ T cells and Tregs, and is required for exhausted T cell regulation (Ruffo et al., 2019). These findings suggest that the epithelial cell subset with the lowest *TP53* score has the highest proliferative capacity and stronger interaction with immunosuppressive ligands at the level of single-cell transcriptomics.

However, it should be noted that this study has some limitations. First, only a limited amount of dataset was used for the performance assessment; therefore, it is necessary to collect additional robust datasets to validate this signature in the future. Second, the biological function of the *TP53*-related gene signature, especially its association with immune infiltration, must be evaluated *via* functional assays.

## Conclusion

This study established and validated a gene signature score derived from *TP53* mutation status, which exhibited prognostic significance for patients with BLCA. The low *TP53* score group was shown to have inactive TP53 and frequent *TP53* mutations, which may suppress antitumor immune signaling and enhance stem cell proliferation signaling. Furthermore, multiple single-cell technologies have characterized the heterogeneity of the tumor immune microenvironment, and a significant enrichment of suppressive Tregs was first revealed in the low *TP53* score group, where the underlying mechanism might be an extensive and dynamic crosstalk between cancer and immunosuppressive cells. These findings identify the *TP53* mutation-based score as a predictive factor for immune response, thereby facilitating the development of new strategies for immunotherapy and treatment monitoring in patients with BLCA.

## Data Availability

The original contributions presented in the study are publicly available. This data can be found here: https://www.ncbi.nlm.nih.gov/, GSE216037.

## Supplementary material

SUPPLEMENTARY FIGURE S1
**(A-B)** Heatmaps showed the expression of immune cells in high and low *TP53* score groups. **(C–F)** Heatmaps and box plots showed the expression of stemness-related gene set in high and low *TP53* score groups.

SUPPLEMENTARY FIGURE S2 The t-**SNE** plot shows the 15 cell subsets after secondary clustering.

SUPPLEMENTARY FIGURE S3
**(A-B)** Distribution of 15 cell subsets in the tumor region and tumor-stromal boundary region.

SUPPLEMENTARY FIGURE S4
**(A-J)** The neighbors of Cancer 1, Cancer 3, CD4T cell, T&B cell and CD47+PD1+ cell s in the high and low *TP53* score groups.

SUPPLEMENTARY FIGURE S5
**(A)** Heatmap shows the expression of marker genes in specified cell subsets. The top bar graph marks a specific cell subset. **(B)** In the cohort of BLCA treated with ICI, the total survival time was grouped according to *TP53* score (high TP53 score and low TP53 score).

SUPPLEMENTARY TABLE S1 TP53-induced and repressed genes symbols.
