# Supplementary material for: TP53-related signature for predicting prognosis and tumor microenvironment characteristics in bladder cancer: A multi-omics study
Source: Front Genet. 2022 Dec 9;13:1057302. doi: 10.3389/fgene.2022.1057302 (PMC9780475; doi:10.3389/fgene.2022.1057302)
Supplement: Supplementary file 1 [file Table1.docx]

**Table S1. Purified antibodies of immune panel**

| **Antibodies** | **Label** | **Clone** | **Source** | **Product_ID** |
| --- | --- | --- | --- | --- |
| Granzyme B | 164Dy | QA18A28 | BioLegend | 396402 |
| HLA-DR | 174Yb | L243 | BioLegend | 307651 |
| TIM-3 | 154Sm | F38-2E2 | BioLegend | 345019 |
| EOMES | 150Nd | 644730 | R&D | MAB6166 |
| Foxp3 | 159Tb | PCH101 | Thermo | 14-4776-82 |
| CEACAM-6 | 145Nd | 439424 | R&D | MAB3934 |
| HLA-DR | 174Yb | L243 | BioLegend | 307651 |
| T-bet | 161Dy | 4B10 | BioLegend | 644825 |
| TCR | 146Nd | B1 | BioLegend | 331202 |
| TNF-α | 158Gd | MAb11 | BioLegend | 502902 |
| CD3 | 170Er | UCHT1 | BioLegend | 300402 |
| CD4 | 176Yb | RPA-T4 | BioLegend | 300541 |
| CD7 | 162Dy | CD7-6B7 | BioLegend | 343111 |
| CD8a | 168Er | RPA-T8 | BioLegend | 301053 |
| CD11b | 172Yb | ICRF44 | BioLegend | 301302 |
| CD11c | 160Gd | Bu15 | BioLegend | 337221 |
| CD14 | 144Nd | M5E2 | BioLegend | 301843 |
| CD19 | 171Yb | HIB19 | BioLegend | 302247 |
| CD20 | 142Nd | 2H7 | BioLegend | 302302 |
| CD27 | 167Er | O323 | BioLegend | 302839 |
| CD28 | 152Sm | CD28.2 | BioLegend | 302937 |
| CD33 | 163Dy | WM53 | BioLegend | 303419 |
| CD45 | 169Tm | HI30 | BioLegend | 304045 |
| CD45RA | 153Eu | HI100 | BioLegend | 304143 |
| CD45RO | 141Pr | UCHL1 | BioLegend | 304202 |
| CD56 | 155Gd | 5.1H11 | BioLegend | 362502 |
| CD68 | 175Lu | Y1/82A | BioLegend | 333802 |
| CD95 | 148Nd | DX2 | BioLegend | 305631 |
| CD127 | 143Nd | A019D5 | BioLegend | 351337 |
| CD137 | 156Gd | 4B4-1 | BioLegend | 309811 |
| CD152 (CTLA-4) | 166Er | L3D10 | BioLegend | 349902 |
| CD163 | 165Ho | GHI/61 | BioLegend | 333602 |
| CD274 (PD-L1) | 149Sm | 29E.2A3 | BioLegend | 329719 |
| CD278 (ICOS) | 151Eu | C398.4A | BioLegend | 313502 |
| CD279 (PD-1) | 147Sm | EH12.2H7 | BioLegend | 329941 |

**Table S2. Purified antibodies of IMC pane**

| **Antibodies** | **Label** | **Clone** | **Source** | **Product_ID** |
| --- | --- | --- | --- | --- |
| Alpha-Smooth Muscle Actin | 142Nd | N terminal | Abcam | ab5694 |
| Beta-catenin | 165Ho | D13A1 | Fluidigm | 3165032D |
| CD90 | 163Dy | 7E1B11 | Abcam | ab181469 |
| Collagen I | 169Tm | **Polyclonal** | Fluidigm | 3169023D |
| Pan-CK | 160Gd | c-11 | Abcam | ab7753 |
| CD326/EpCAM | 150Nd | aa 250 to the C-terminus | Abcam | ab71916 |
| CD44 | 153Eu | 692-742 | Abcam | ab157107 |
| VEGFR-2 | 175Lu | phosphorylation site of tyrosine 951 | Abcam | ab39638 |
| FAP | 147Sm | DPPIV/CD26 | Abcam | ab53066 |
| CD31 | 156Gd | JC/70A | Abcam | ab9498 |
| E-cadherin | 158Gd | 24E10 | Fluidigm | 3158029D |
| Vimentin | 143Nd | RV202 | Fluidigm | 3143029D |
| YAP1 | 149Sm | 53-162 | Abcam | ab56701 |
| CD66a | 171Yb | CD66a-B1.1 | Fluidigm | 3171020D |
| Ki-67 | 168Er | B56 | Fluidigm | 3168022D |
| CK-5 | 164Dy | Cytokeratin 5 | Abcam | ab53121 |
| SNAIL+SLUG | 151Eu | aa 236-264 | Abcam | ab180714 |
| ER-β | 174Yb | 14C8 | Abcam | ab288 |
| CD133 | 172Yb | aa 848-865 | Abcam | ab16518 |
| Foxp3 | 155Gd | 43-100 | NOVUS | NB100-39002 |
| PD_L1 | 145Nd | 73-10 | Abcam | ab226766 |
| AR | 166Er | AR 44 | Abcam | ab9474 |
| CTLA4 | 173Yb | CAL49 | Abcam | ab237712 |
| PD-1 | 154Gd | CAL20 | Abcam | ab201811 |
| ALDH | 146Nd | 44/ALDH | BD | 611195 |
| CD47 | 144Nd | Gln19-Pro139 | R&D | AF4670 |
| TGF-β1 | 167Er | TB21 | Abcam | ab190503 |
| CD68 | 141Pr | KPI | Abcam | ab213096 |
| CD4 | 159Tb | RPA-T4 | Biolegend | 300502 |
| CD8a | 162Dy | C8/144B | Fluidigm | 3162034D |
| CD20 | 161Dy | H1 | Fluidigm | 3161029D |
| CD3 | 170Er | C-Terminal | Fluidigm | 3170019D |
| CD45 | 152Sm | CD45-2B11 | Fluidigm | 3152016D |
