# Supplementary figures and images for: TP53-related signature for predicting prognosis and tumor microenvironment characteristics in bladder cancer: A multi-omics study
Source: Front Genet. 2022 Dec 9;13:1057302. doi: 10.3389/fgene.2022.1057302 (PMC9780475; doi:10.3389/fgene.2022.1057302)

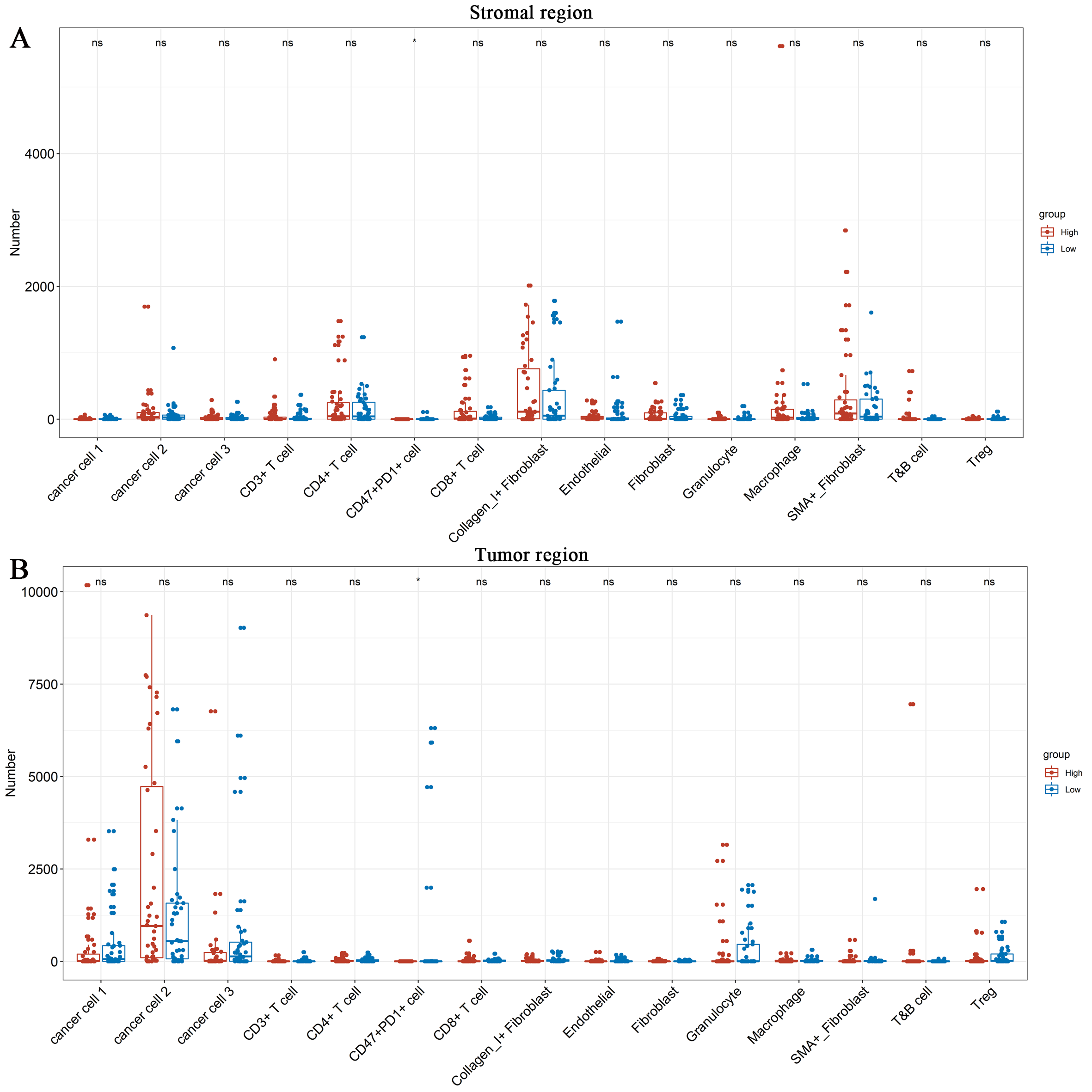

Supplement: Supplementary file 2 [file Image3.TIF]

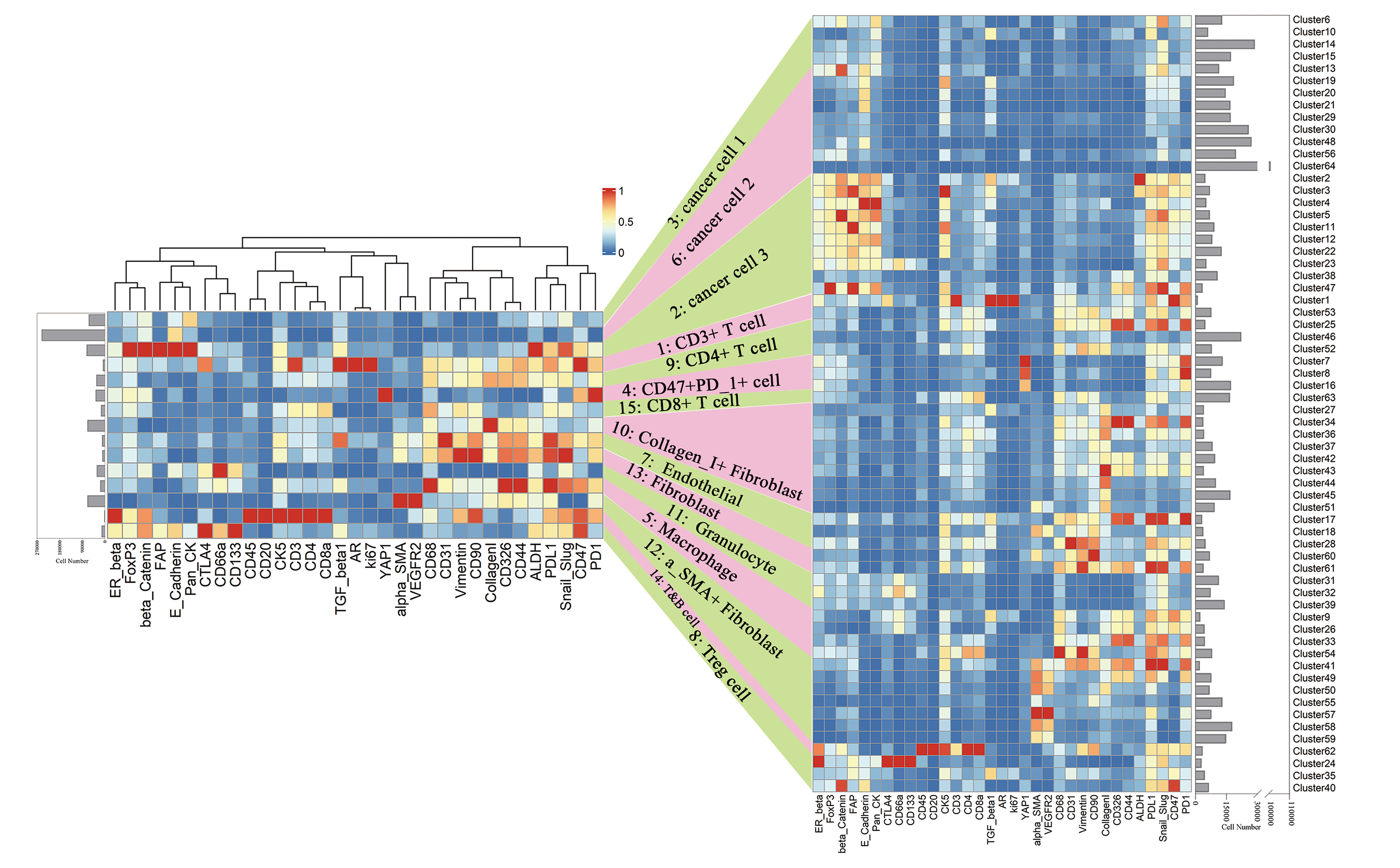

Supplement: Supplementary file 4 [file Image2.TIF]

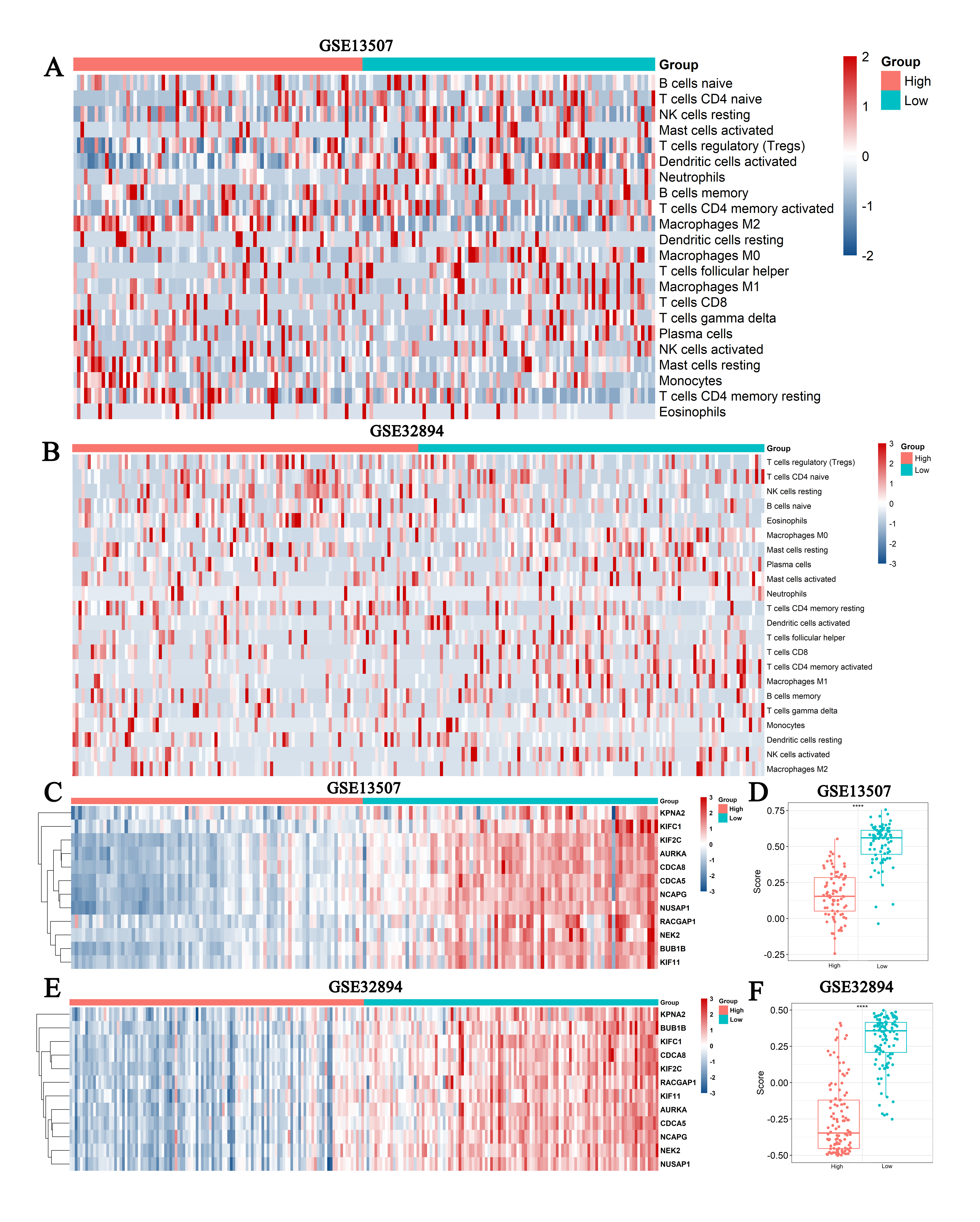

Supplement: Supplementary file 5 [file Image1.TIF]

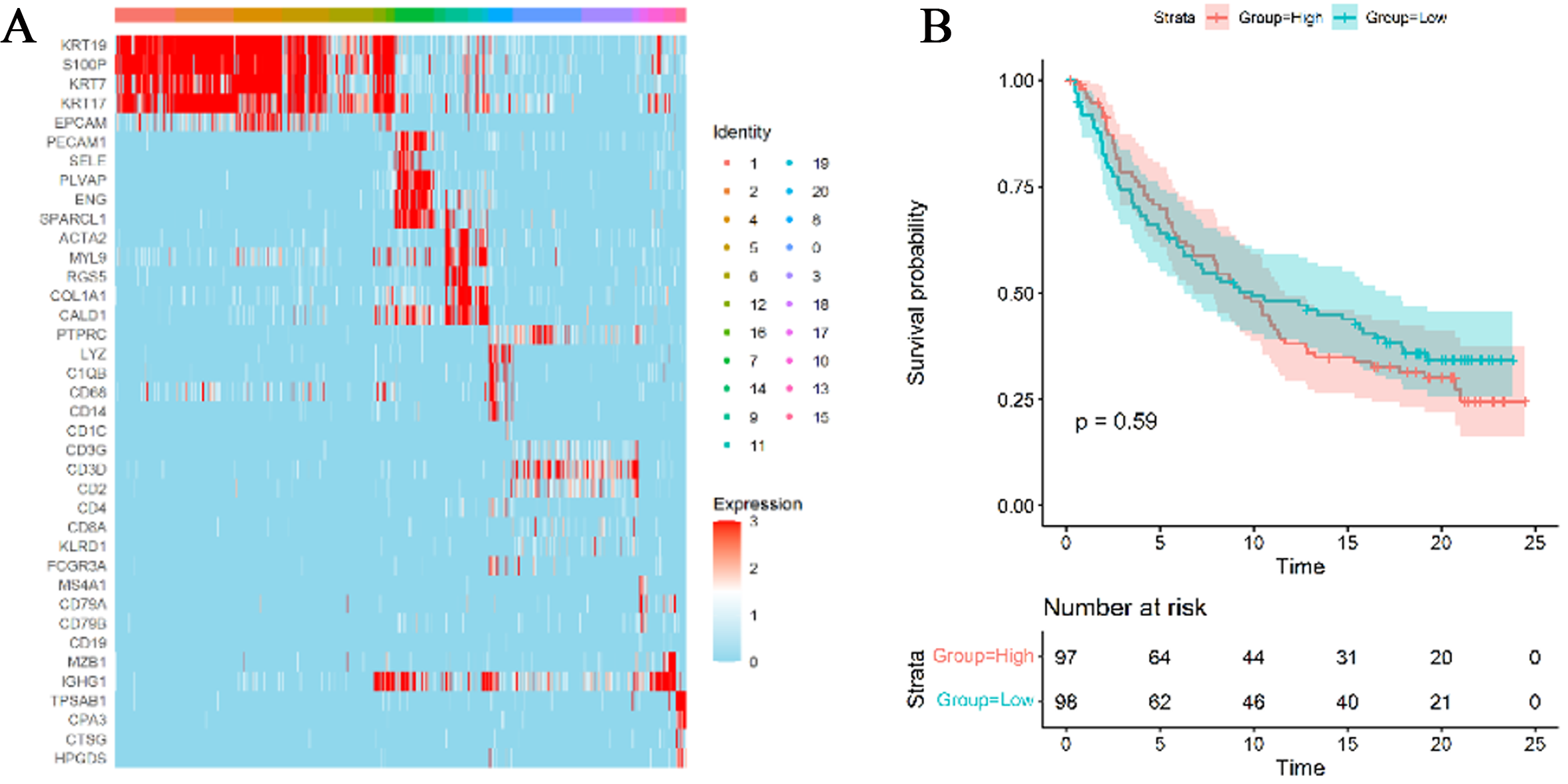

Supplement: Supplementary file 6 [file Image5.TIF]
